# First Report of *Longidorus Leptocephalus* Hooper, 1961 (Nematoda: Longidoridae) from Greece

**DOI:** 10.2478/jofnem-2022-0027

**Published:** 2022-07-29

**Authors:** Ilenia Clavero-Camacho, Carolina Cantalapiedra-Navarrete, Maria Kormpi, Juan E. Palomares-Rius, Emmanuel A. Tzortzakakis, Pablo Castillo, Antonio Archidona-Yuste

**Affiliations:** 1 Institute for Sustainable Agriculture (IAS), CSIC, Avenida Menéndez Pidal s/n, Campus de Excelencia Internacional Agroalimentario, ceiA3, 14004, Córdoba, Spain; 2 Benaki Phytopathological Institute, Kifisia, Athens, Greece; 3 Department of Viticulture, Vegetable Crops, Floriculture and Plant Protection, Institute of Olive Tree, Subtropical Crops and Viticulture, ELGO-DIMITRA, Mesa Katsabas, 71307, Heraklion, Crete, Greece; 4 Andalusian Institute of Agricultural and Fisheries Research and Training (IFAPA), Centro Alameda del Obispo, 14004, Córdoba, Spain

**Keywords:** cytochrome oxidase c subunit 1, D2-D3 of 28S rDNA, description, ITS1 rDNA, needle nematodes, taxonomy

## Abstract

Longidorid nematodes comprise more than 500 species, and *Longidorus* and *Xiphinema* are the most diversified, prevalent, and cosmopolitan genera within plant-parasitic nematodes. The genus *Longidorus* comprise a group of species, some of which are vectors of plant viruses. New sampling for needle nematodes was carried out in a grapevine area in Thessaloniki, northern Greece, and one nematode species of *Longidorus* (*L. leptocephalus*) was recovered. Nematodes were extracted from soil samples by modified sieving and a decanting method. Extracted specimens were processed using glycerol, mounted on permanent slides, and subsequently identified morphologically. Nematode DNA was extracted from individual, live specimens, and PCR (Polymerase Chain Reaction) assays were performed for D2-D3 expansion segments of 28S rRNA, ITS1 rRNA, and partial mitochondrial *COI* regions. Morphology and morphometric data obtained from this population were consistent with the original description and reported populations of *L. leptocephalus*. To our knowledge, this is the first report of *L. leptocephalus* in Greece and the second in the Mediterranean Basin after the record of the species from Slovenia, extending the geographical distribution of this species in Europe.

Needle nematodes belonging to the genus *Longidorus* Micoletzky, 1922 are polyphagous root ectoparasites parasitizing a wide range of economically important plants by directly feeding on root cells. Some species of this genus are economically important pests of agricultural plants, and some are proved to transmit nepoviruses (Taylor and Brown, 1997). The genus consists of more than 160 valid species (Archidona-Yuste et al., 2016; Gharibzadeh et al., 2018; Cai et al., 2020, Bakhshi Amrei et al., 2022). Currently, 14 *Longidorus* spp. have been reported in Greece, but only nine of these have been molecularly identified: *Longidorus closelongatus* Stoyanov, 1964, *L. cretensis* Tzortzakakis et al., 2001, *L. euonymus* Mali and Hooper, 1974, *L. iranicus* (= *moesicus*) Sturhan and Barooti, 1983, *L. kuiperi* Brinkman et al., 1987, *L. orientalis* Loof, 1982, *L. pisi* (= *latocephalus*) Edward et al., 1964, *L. pseudoelongatus* Altherr, 1976, and *L. pauli* Lamberti et al., 1999 (He et al., 2005; Tzortzakakis et al., 2014, 2016, 2017, 2021). For the five remaining species *L. africanus* Merny, 1966, *L. elongatus* (de Man, 1876) Micoletzky, 1922,

*L. fasciatus* Roca and Lamberti, 1981, *L. intermedius* Kozlowska and Seinhorst, 1979, and *L. proximus* Sturhan and Argo, 1983, there is currently no molecular data available (Tzortzakakis et al., 2008). During a recent nematode sampling from a grapevine area in northern Greece, a needle nematode population was detected resembling *L. leptocephalus* Hooper, 1961.

*Longidorus leptocephalus* has been reported in several countries from central and northern Europe, including Austria, Belgium, Bulgaria, Czech Republic, Denmark, Germany, Ireland, Netherlands, Norway, Poland, Russia and Sweden (Boag and Brown, 1976; Brown and Taylor, 1987; Peneva and Choleva, 1994; Tiefenbrunner and Tiefenbrunner, 2004; Kumari and Decraemer, 2007), and north-eastern Slovenia (Širca et al., 2007). This nematode has been associated with wild plants (i.e., forests, wild rose) and several fruit crops (viz. grapevine, fruit orchards) (Brown and Taylor, 1987). Although *L. leptocephalus* has been reported as a virus vector of the English strain of raspberry ringspot virus (Valdez, 1972), this species does not fulfill the criteria for virus vectors as required by Trudgill et al. (1983). Similarly, Flegg (1969) found that *L. leptocephalus* in the rhizosphere of cherry trees was infected with cherry leaf roll virus, but they did not do any laboratory tests of virus transmission on it. Therefore, the objective of the present study was to provide an accurate identification of the needle nematode population detected in Thessaloniki by an integrative taxonomic approach using morphological and molecular data based on D2-D3 expansion segments of 28 and ITS1 rDNA, as well as partial mitochondrial *COI* regions.

## Materials and Methods

### Nematode samples and morphological study

Soil samples containing needle nematodes resembling *L. leptocephalus* were collected at a depth of 10 to 30 cm from the rhizosphere of grapevine in the area of Thessaloniki, northern Greece. Nematodes were extracted from soil by modified sieving and a decanting method (Brown and Boag, 1988). Extracted specimens were handpicked, heat killed, fixed in TAF (triethanolamine formalin), processed using glycerol by a slow evaporation method, and mounted on permanent slides (Hooper, 1986). The light micrographs and measurements of nematodes population, including the main diagnostic characteristics, were performed using a Leica DM6 compound microscope with a Leica DFC7000 T digital camera. All other abbreviations used were as defined in the study by Jairajpuri and Ahmad (1992).

### Molecular characterization

For molecular analyses and in order to avoid mistakes in case of potential mixed populations in the same sample, single specimens from the sample were temporarily mounted in a drop of 1 M NaCl containing glass beads (to avoid nematode crushing/ damaging specimens) to ensure that specimens were conformed with the target population. All necessary morphological and morphometric data were recorded. This was followed by DNA extraction from single individuals as described by Archidona-Yuste et al. (2016). The D2-D3 segments were amplified using the D2A (5'-ACAAGTACCGTGAGGGAAAGTTG-3') and D3B (5'-TCGGAAGGAACCAGCTACTA-3') primers (De Ley et al., 1999). The internal transcribed spacer region 1 (ITS1) was amplified using forward primer 18S (5'-TTGATTACGTCCCTGCCCTTT-3') (Vrain et al., 1992) and reverse primer rDNA1 5.8S (5'-ACGAGCCGAGTGATCCACCG-3') (Cherry et al., 1997). Finally, the portion of the *COI* gene was amplified as described by Lazarova et al. (2006) using the primers COIF (5'-GATTTTTTGGKCATCCWGARG-3') and COIR (5'-CWACATAATAAGTATCATG-3').

All PCR assays were done according to the conditions described by Archidona-Yuste et al. (2016). Then, the amplified PCR products were purified using ExoSAP-IT (Affymetrix, USB products) and used for direct sequencing on a DNA multicapillary sequencer (Model 3130XL genetic analyzer; [Applied Biosystems Foster City, CA]), using the BigDye Terminator Sequencing Kit V.3.1 (Applied Biosystems, Foster City, CA), at the Stab Vida sequencing facilities (Caparica, Portugal). The newly obtained sequences were submitted to the GenBank database under the accession numbers indicated on the phylogenetic trees. Voucher specimens of the studied population were deposited in the nematode collection of the Institute for Sustainable Agriculture, IAS-CSIC, Córdoba, Spain.

### Phylogenetic analyses

D2-D3 expansion segments of 28S and ITS1 rDNA as well as *COI* mtDNA sequences of the recovered *Longidorus* population were obtained in this study. These newly generated sequences, and other sequences of *Longidorus* spp. retrieved from GenBank, were used for phylogenetic analyses. Outgroup taxa for each dataset were chosen following previously published studies (He et al., 2005; Archidona-Yuste *et al.,* 2019; Bakhshi Amrei et al., 2022). Multiple sequence alignments of the three aforementioned loci were made using the FFT-NS-2 algorithm of MAFFT V.7.450 (Katoh et al., 2019). The alignments were manually visualized using BioEdit (Hall, 1999) and edited by Gblocks ver. 0.91b (Castresana, 2000) in the Castresana Laboratory server (http://molevol.cmima.csic.es/castresana/Gblocks_server.html) using options for a less stringent selection (minimum number of sequences for a conserved or a flanking position: 50% of the number of sequences +1; maximum number of contiguous nonconserved positions: 8; minimum length of a block: 5; allowed gap positions: with half). Phylogenetic analyses of the sequence datasets were based on Bayesian inference (BI) using MrBayes 3.1.2 (Ronquist and Huelsenbeck, 2003). The best-fit model of DNA evolution was obtained using JModelTest V.2.1.7 (Darriba et al., 2012) with the Akaike information criterion (AIC). The best-fit model, the base frequency, the proportion of invariable sites, and the gamma distribution shape parameters and substitution rates in the AIC were then used in MrBayes for the phylogenetic analyses. The general time-reversible model with invariable sites and a gamma-shaped distribution (GTR  +  I +  G) for the D2-D3 segments of 28S rDNA, GTR + G model for ITS1 rDNA, and the transitional model and a gamma-shaped distribution (TIM3 + G) for the partial *COI* gene and all three datasets were run with four chains for 4 ´ 10^6^ generations. A combined analysis of the two ribosomal genes was not undertaken due to some sequences not being available for all species. The Markov chains were sampled at intervals of 100 generations. After discarding burn-in samples of 30% and evaluating convergence, the remaining samples were retained for in-depth analyses. The topologies were used to generate a 50% majority-rule consensus tree. Posterior probabilities (PP) were given on appropriate clades. Trees from all analyses were visualized using FigTree software, version v.1.42 (Rambaut, 2014).

## Results

**Figure 1:**
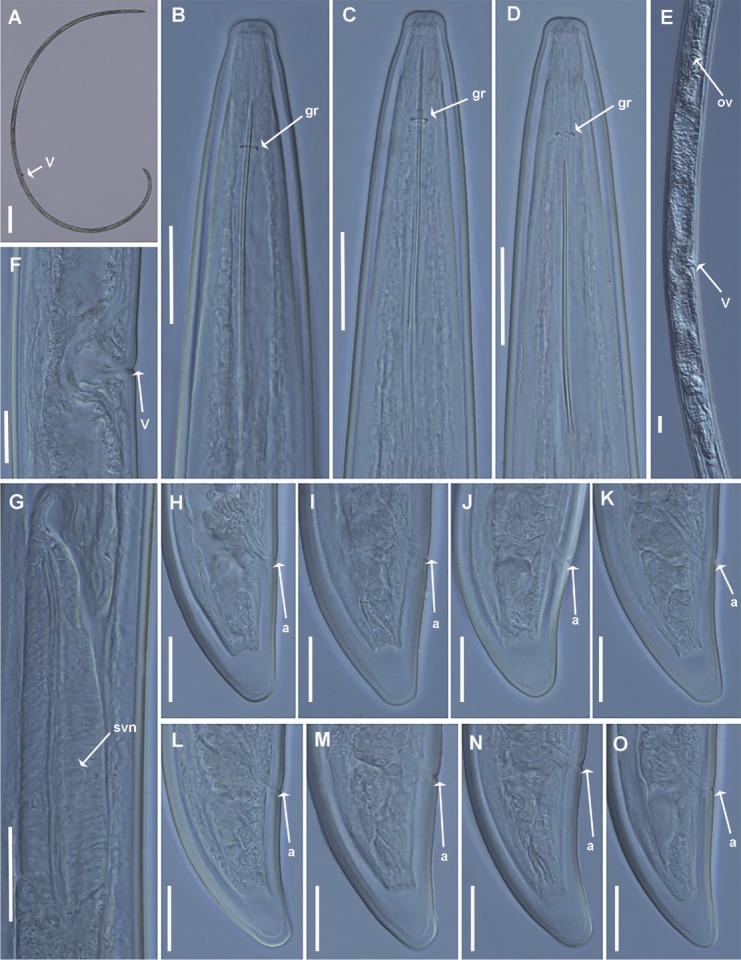
Light micrographs of *Longidorus leptocephalus* Hooper, 1961 from Greece (A–O). (A) Whole female, (B–D) female anterior regions, (E) detail of female reproductive system, (F) vulval region, (G) detail of basal bulb, and (H–O) female tail regions. a, anus; gr, guiding ring; ov, ovary; svn, ventrosublateral nucleus; V, vulva (Scale bars: A = 200 mm, B–O = 20 mm).

Soil samples from grapevine (rootstock, 1103 Paulsen) at Thessaloniki yielded a parthenogenetic *Longidorus* population resembling *L. leptocephalus* with a density of ca. 40 needle nematodes/kg of soil (Figure 1).

Greek population of *Longidorus leptocephalus* Hooper, 1961.

### Measurements

See Table 1.

**Table 1:** Morphometrics of *Longidorus leptocephalus* Hooper, 1961 from Greece and type population.

	Thessaloniki, Greece	Highfield, Scotland
Character^a^	Females	Female paratypes
N	10	16
L (mm)	4.9 ± 0.43 (4.25–5.53)	4.2 (3.6–4.6)
A	99.9 ± 8.1 (90.1–113.0)	96.0 (83.0–121.0)
B	14.3 ± 1.6 (12.2–17.3)	12.0 (11.7–12.4)
C	110.2 ± 17.4 (83.4–131.7)	96.0 (78.0–105.0)
c’	1.4 ± 0.1 (1.2–1.5)	1.25
d^b^	2.8 ± 0.1 (2.6–2.9)	–
d’^c^	2.0 ± 0.1 (1.9–2.1)	–
V	49.0 ± 2.9 (45.1–55.0)	54.0 (52.0–57.0)
Odontostyle length	65.4 ± 4.3 (58.0–69.5)	64 (60–70)
Odontophore length	44.9 ± 5.0 (38–52)	46 (41–56)
Total stylet length	110.3 ± 8.3 (96–118)	–
Anterior end to guiding ring	26.9 ± 1.0 (25.0–28.5)	30 (29–32)
Hyaline part of tail	14.7 ± 1.7 (12–17)	–
Body width at level of:		
lip region	9.7 ± 0.4 (9–10)	–
guiding ring	19.0 ± 0.4 (18.0–19.5)	–
Anus	32.5 ± 1.7 (29–35)	–
Tail length	44.8 ± 3.9 (41.5–51.0)	–

Measurements in mm, at exception of L in mm. ^a^Abbreviations are defined in the study by Jairajpuri and Ahmad (1992). ^b^d = anterior to the guide ring/body width at the lip region (Brown et al., 1994). ^c^d’ = body width at the guide ring/body width at the lip region (Brown et al., 1994).

## Description

### Female

Medium sized. Body habitus ventrally curved in a close C-shaped to single spiral when killed by gentle heat. Cuticle 2.9 ± 0.3 (2.6–3.2) mm thick at mid body. Lip region narrow and rounded, slightly offset by a depression from the rest of body, half body-width at the level of guiding ring. Amphidial pouches asymmetrically bilobed at base. Guiding ring single, at 2.6–2.9 times lip region diameter distance from anterior end. Odontostyle 1.3–1.7 times as long as odontophore; odontophore with slight basal swellings. Pharynx extending to a terminal pharyngeal bulb (87.7 ± 7.7 [80.0–103.0] μm long), with dorsal gland nucleus (DN) and ventrosublateral gland nuclei (SVN) separately located at 16.4 (11.9–19.8)% and 59.4 (52.9–67.1)% of distance from anterior end of pharyngeal bulb, respectively. Glandularium 77.0 (67.0–87.0) μm long. Cardia conoid-rounded. Vulva at about middle of the body. Vagina 13.3 (11.0–16.0) mm long, ovejector 25.3 (21.5–28.0) mm wide. Genital system amphidelphic, anterior and posterior genital branches almost equally developed, 237–568 and 260–554 mm long, respectively. Sperm cells not detected inside uterus or oviduct. Rectum 22.2 (19.0– 26.0) mm long. Tail conical, dorsally convex, ventrally concave to flat with bluntly rounded terminus.

### Male

Not found.

### Remarks

According to the polytomous key by Chen et al. (1997), and supplement by Loof and Chen (1999), the Greek population has the following codes (codes in parentheses are exceptions): A2(1) – B1 – C2 – D3 – E1 – F2(3) – G2– H4 – I1.

Up to our knowledge, the Greek population of *L. leptocephalus* collected from the rhizosphere of grapevine at Thessaloniki was the first report of this species from Greece and the second from the Mediterranean Basin. Morphology and morphometrics of this Greek population fit the type population (Hooper, 1961) and other populations from Europe (Lišková and Brown, 1995; Kumari and Decraemer, 2007; Širca et al., 2007). Body and odontostyle lengths of the Greek population of the species agreed with those by Flegg (1967) and Lišková and Brown (1995) identified as “small form” having a 60–70 μm long odontostyle. However, considering the great cryptic diversity within longidorids (Cai et al., 2020), an integrative taxonomical analysis should be carried out also on “large forms” characterized by having 70 to 80 μm long odontostyle to better confirm the species identification (Flegg, 1967; Hooper, 1973). The minor differences between Greek population and the type population of *L. leptocephalus* are as follows: slightly longer body (4.87 [4.25–5.53] mm vs 4.2 [3.6–4.6] mm), and wider c’ ratio range (1.4 [1.2– 1.5] vs 1.25). These small morphometric differences widen the range of morphometric data and may be due to geographical intraspecific variations.

Type population of *Longidorus leptocephalus* is morphologically and morphometrically close to type population of *L. aetnaeus* Roca et al., 1986, especially in the shape of the lip and tail regions, but differs in body (2.69–3.68 mm) and odontostyle lengths (72– 80 μm), position of the vulva (44–47), and presence/ absence of males. Furthermore, both species can be also separated by molecular markers, i.e., by differences in 28S and ITS1 rDNA and *COI* mtDNA sequences (see below).

## Molecular Characterization and Phylogeny of *Longidorus leptocephalus* from Greece

Amplification and sequencing of the D2-D3 expansion domains of 28S, ITS1 rDNA, and partial *COI* genes yielded sequences of *ca* 900 bp, 1,100 bp, and 400 bp, respectively, based on gel electrophoresis. Four D2-D3 of 28S rDNA (ON241755-ON241758), three ITS1 (ON241759-ON24175961), and four partial *COI* sequences (ON142397-ON142400) were generated for this population without intraspecific sequence variations, except for the *COI* where one variable position was detected. All of D2-D3 sequences were found to be identical to several accessions from *L. leptocephalus* deposited in GenBank such as DQ364600, KF242325, KF242326, and KF242327. COI sequences were shown to be 94% identical to the accession number KY816682 (differing from 16 to 17 nucleotides). ITS sequences from *L. leptocephalus* were generated the first time in this study. The closest species to *L. leptocephalus* was *L. aetnaeus*, while BLAST search, being 99% identical for the D2-D3 region (KC357771) (differing from 6 nucleotides and 1 indel), and 82% identical to *COI* gene (ON142397-ON142400) (differing from 51 to 52 nucleotides and no indels). No ITS data are available for *L. aetnaeus* for comparison.

**Figure 2:**
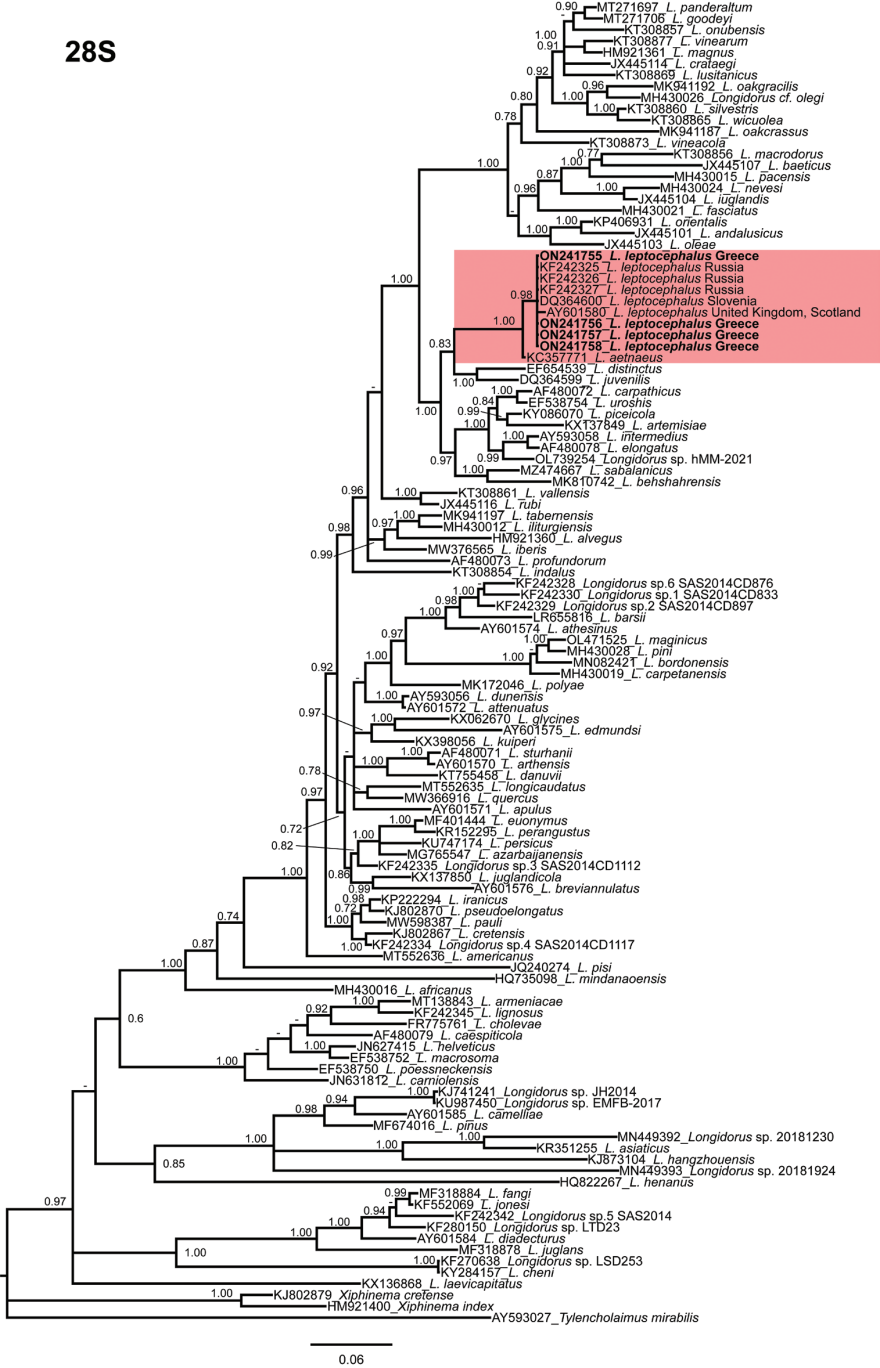
Phylogenetic relationships of *Longidorus leptocephalus* Hooper, 1961 within the genus *Longidorus*. Bayesian 50% majority rule consensus tree as inferred from D2 and D3 expansion domains of 28S rDNA sequence alignment under the general time-reversible model of sequence evolution with correction for invariable sites and a gamma-shaped distribution (GTR + I + G). Posterior probabilities more than 0.70 are given for appropriate clades. Newly obtained sequences in this study are shown in bold. Scale bar = expected changes per site. PP. posterior probabilities.

**Figure 3:**
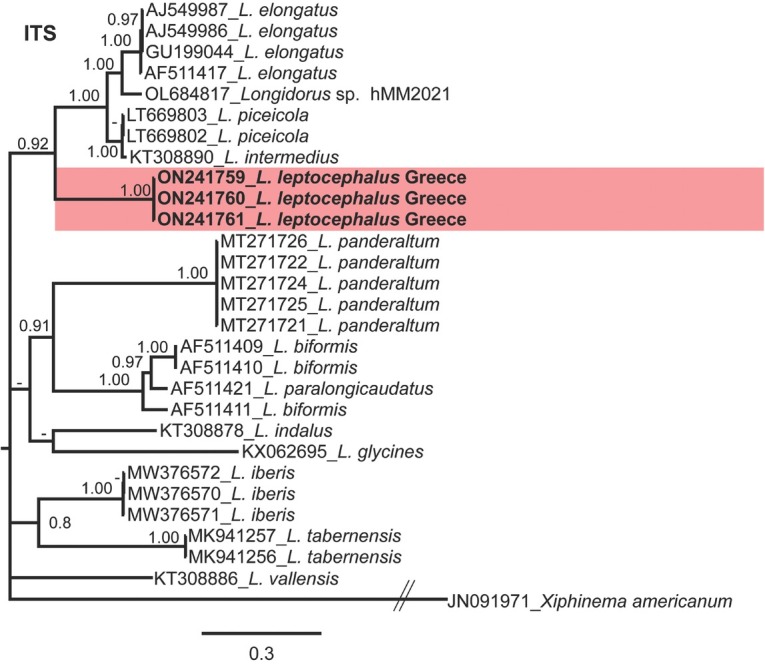
Phylogenetic relationships of *Longidorus leptocephalus* Hooper, 1961 from Greece within the genus *Longidorus*. Bayesian 50% majority rule consensus tree as inferred from ITS1 rDNA sequence alignment under the general time-reversible model of sequence evolution and a gamma-shaped distribution (GTR + G). Posterior probabilities more than 0.70 are given for appropriate clades. Newly obtained sequences in this study are shown in bold. Scale bar = expected changes per site. PP, posterior probabilities.

**Figure 4:**
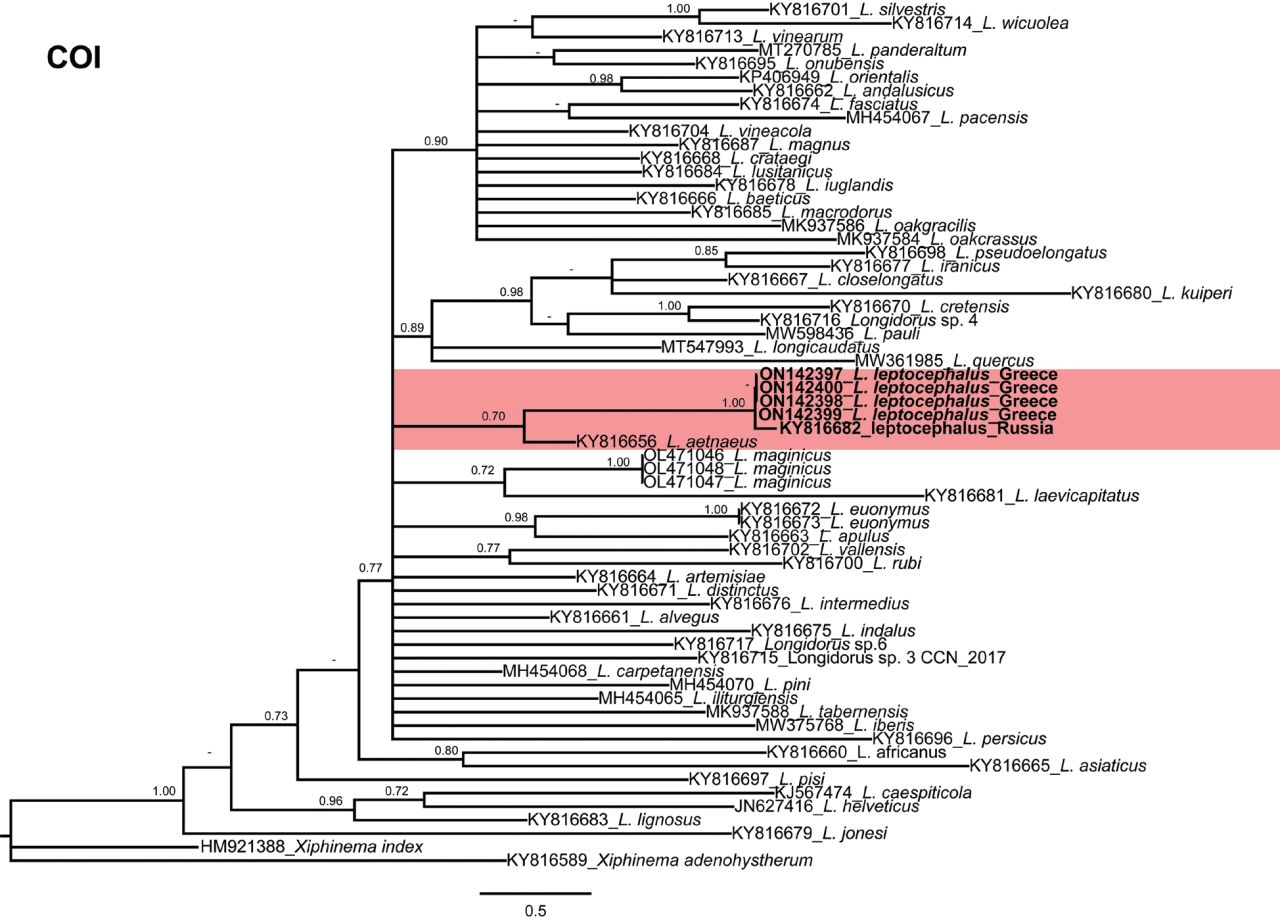
Phylogenetic relationships of *Longidorus leptocephalus* Hooper, 1961 from Greece within the genus *Longidorus*. Bayesian 50% majority-rule consensus trees as inferred from cytochrome c oxidase subunit I (*COI* mtDNA gene) sequence alignments under the TIM1 + G model. Posterior probabilities more than 0.70 are given for appropriate clades. Newly obtained sequences in this study are in bold letters. PP, posterior probabilities.

Phylogenetic relationships among *Longidorus* species, as inferred from analyses of D2-D3 expansion domains of 28S, ITS1 rDNA, and the partial *COI* mtDNA gene sequences using BI, are shown in Figures 2–4, respectively. The phylogenetic trees generated with the ribosomal and mitochondrial DNA markers included 117, 29, and 64 sequences with 749, 1019, and 389 characters in their alignments, respectively.

In 28S phylogeny, *Longidorus leptocephalus* formed a clade with clustered with *L. aetnaeus* (PP = 1.00). Their clade is in sister relation with the clade including *L. elongatus*, *L. distinctus* Lamberti et al., 1983, *L. intermedius*, *L. piceicola* Lisková et al., 1997, *L. juvenilis* Dalmasso, 1969, *L. carpathicus* Lisková et al., 1997, *L. uroshis* Krnjaic et al., 2000, *L. artemisiae* Rubtsova et al., 1999, *L. sabalanicus* Asgari et al., 2022, and *L. behshahrensis* Bakhshi Amrei et al., 2020. In *COI* phylogeny, *Longidorus leptocephalus* formed a clade with *L. aetnaeus* (PP = 0.70). Since poor identity/coverage was detected for newly generated ITS1 sequences of *L. leptocephalus* from Greece (ON241759-ON241761) with other sequences available in GenBank, only the sequences having high coverage were included in the analyses of this region (Bakhshi Amrei et al., 2022). The 50% majority rule consensus ITS1 BI tree (Fig. 3) showed a moderate supported clade (PP = 0.92) including *L. leptocephalus* (ON241759-ON241761) and *L. elongatus* (AJ549986-AJ549987, GU199044 and AF511417), *L. intermedius* (KT308890), and *L. piceicola* (LT669802-LT669803). These species were also phylogenetically related to *L. leptocephalus* when the D2-D3 region was used.

The herein resolved phylogenies based on two rDNA molecular markers (D2-D3 expansion domains of 28S and ITS1 rDNA regions) and *COI* mtDNA were generally congruent with those presented by previous authors (Gutiérrez-Gutiérrez et al., 2013; Archidona-Yuste et al., 2019; Cai et al., 2020; Clavero-Camacho et al., 2021; Inserra et al., 2021; Bakhshi Amrei et al., 2022).

This research widens the number of *Longidorus* species occurring in Greece, as well as the molecular data of *Longidorus*. This is one of the cases of which two morphologically close species (Bakhshi Amrei et al., 2013) have close phylogenetic affinities using ribosomal and mitochondrial sequences as well.

In conclusion, the present study increases the prodigious biodiversity of this genus in the Mediterranean Basin by adding a new record.
